# Persulfate Oxidation Coupled with Biodegradation by *Pseudomonas fluorescens* Enhances Naphthenic Acid Remediation and Toxicity Reduction

**DOI:** 10.3390/microorganisms9071502

**Published:** 2021-07-14

**Authors:** Amy-lynne Balaberda, Ania C. Ulrich

**Affiliations:** Department of Civil & Environmental Engineering, University of Alberta, Edmonton, AB T6G 1H9, Canada; balaberd@ualberta.ca

**Keywords:** oilsands, bioremediation, oxidation, persulfate, tailings, naphthenic acids

## Abstract

The extraction of bitumen from the Albertan oilsands produces large amounts of oil sands process-affected water (OSPW) that requires remediation. Classical naphthenic acids (NAs), a complex mixture of organic compounds containing O_2_^−^ species, are present in the acid extractable organic fraction of OSPW and are a primary cause of acute toxicity. A potential remediation strategy is combining chemical oxidation and biodegradation. Persulfate as an oxidant is advantageous, as it is powerful, economical, and less harmful towards microorganisms. This is the first study to examine persulfate oxidation coupled to biodegradation for NA remediation. Merichem NAs were reacted with 100, 250, 500, and 1000 mg/L of unactivated persulfate at 21 °C and 500 and 1000 mg/L of activated persulfate at 30 °C, then inoculated with *Pseudomonas fluorescens* LP6a after 2 months. At 21 °C, the coupled treatment removed 52.8–98.9% of Merichem NAs, while 30 °C saw increased removals of 99.4–99.7%. Coupling persulfate oxidation with biodegradation improved removal of Merichem NAs and chemical oxidation demand by up to 1.8× and 6.7×, respectively, and microbial viability was enhanced up to 4.6×. Acute toxicity towards *Vibrio fischeri* was negatively impacted by synergistic interactions between the persulfate and Merichem NAs; however, it was ultimately reduced by 74.5–100%. This study supports that persulfate oxidation coupled to biodegradation is an effective and feasible treatment to remove NAs and reduce toxicity.

## 1. Introduction

Alberta, Canada has one of the largest oil reserves in the world, where the extraction of surface mined bitumen produces large amounts of oil sands process-affected water (OSPW) that is stored in tailings ponds, along with leftover sand and clay [1,2,3]. While much of the OSPW can be recycled after the sand and clay have settled, this creates an increasingly contaminated waste stream and may reduce the recovery rate of bitumen [3]. There are numerous contaminants present in OSPW such as salts, minerals, heavy metals, bitumen, polycyclic aromatic hydrocarbons (PAHs), benzene, toluene, ethylbenzene, xylene (BTEX), and naphthenic acids (NAs) [3,4].

NAs are organic compounds in bitumen that are solubilized during the extraction process and are considered a primary cause of OSPW acute toxicity [3,5,6]. NAs are classically defined as a complex mixture of cyclic and acyclic alkanes possessing a single carboxylated side chain, with the general formula C_n_H_2n+z_O_2_, where *n* represents the carbon number, and Z represents the number of hydrogen atoms lost due to ring and double bond formation [7]. Naphthenic acid fraction compounds (NAFCs) is a more general term that includes classical NAs (O_2_^−^ species), along with a complex mixture of oxidized compounds (three or more oxygen atoms) and compounds containing sulfur and nitrogen [8]. With the large footprint currently required to store tailings and concerns of subsurface contamination, effective water management and remediation methods is an ever-increasing concern.

There are various technologies being studied for NA removal from OSPW, including: adsorption with activated carbon, membrane filtration (micro/ultra-filtration, nanofiltration, reverse osmosis), coagulation/flocculation, advanced oxidation processes, enhanced biodegradation (via in situ biostimulation or bioreactors), and constructed wetlands or end pit lakes [9,10]. Chemical oxidation, primarily ozonation, has exhibited very promising results; however, numerous studies have shown that increased ozone exposure time does not lead to a proportional increase in NA degradation [11,12,13]. This suggests that the oxidant may not be able to access the residual contaminant, and complete mineralization would be costly for large-scale application [2]. Currently, biodegradation is considered the most cost-effective treatment option for NAs; however, a portion of NAs are resistant to biodegradation by indigenous tailings microorganisms [5,14,15].

A potential strategy that was explored in this study is a combined treatment, where a limited amount of chemical oxidant is applied to breakdown the recalcitrant NA fraction into simpler, more bioavailable compounds for the microorganisms to further degrade. Chemical oxidation can also enhance bioremediation by decreasing the concentration of pollutants to less toxic levels [16,17]. Therefore, coupling chemical oxidation and biodegradation can provide a more economical and efficient remediation method, utilizing the advantages of each, while overcoming their respective disadvantages. The main concern with a combined treatment is how the in situ application of a chemical oxidant will impact the microbial population, which can occur through oxidative stress, changes in pH, and changes in redox conditions [16]; however, there remains little research in this area.

The most common chemical oxidants studied for OSPW treatment include chlorine, hydrogen peroxide, ozone, and permanganate [9,10]. However, only ozone has been studied as a pre-treatment for biodegradation, as ozone is highly reactive with the most bio-persistent cyclic NA fraction [13,18,19,20]. The most frequently used application involves treating ozonated OSPW using ex situ bioreactors that contain a previously established enriched microbial community [19,20,21,22,23,24,25,26]. These studies have shown thicker biofilms and improved NAFC removals (78.8–99.9%) compared to ozonation or biodegradation alone. Microcosm studies have also been conducted, where ozonated OSPW is re-inoculated with a pellet of indigenous OSPW microorganisms, and results have shown improved toxicity reduction [13,18,27,28,29].

Another treatment strategy involves simultaneously coupling chemical oxidation and biodegradation in situ; therefore, it is essential to know how microorganisms will react to the oxidative stress. Brown et al. [30] utilized a coupled system and found that indigenous OSPW microorganisms were not eliminated after ozonation (50 mg/L), effectively re-established, and performed as well as microorganisms not exposed. However, since ozone is an expensive and aggressive oxidant, it may not be an ideal choice for coupling with bioremediation. For more effective overall remediation, it is important to tailor the treatment to the microbial population rather than choosing an oxidant based on the contaminant. Studies have shown that using low doses of a less aggressive oxidant leads to more efficient overall remediation for creosote [31], jet fuel hydrocarbons [32], dinitrotoluene (DNT) [33], and diesel [34].

Recently, sodium persulfate (Na_2_S_2_O_8_), or peroxydisulfate, has been gaining interest as an alternative to ozone (E° = +2.1 V), as it is a powerful oxidant, economical, and possibly less harmful toward microorganisms [33,35,36]. In addition, persulfate has a high water solubility, no odor, is effective over a wide range of pH values, has a low affinity for soil organics, and is persistent [37,38,39].

Persulfate can be used either unactivated (E° = +2.1 V) or activated (E° = +2.6 V) [35]. Unactivated persulfate reacts directly with the compound, taking two electrons to form two sulfate anions (Equation (1)). Activation aims to create sulfate radicals by either imparting energy (heat or ultraviolet light) to cleave the peroxide bond (Equation (2)) or undergoing a redox reaction with an electron donor (transition metals) (Equation (3)) [40]. Hydroxyl radicals (E° = +2.8 V) can also be formed when the sulfate radical reacts with water (Equation (4)) or hydroxyl ions (Equation (5)) [38].

S_2_O_8_^2−^ + 2𝑒^−^ → 2SO_4_^2−^ (1)

S_2_O_8_^2−^ + energy input → 2SO_4_•^−^ (2)

S_2_O_8_^2−^ + M^n+^ → SO_4_•^−^ + SO_4_^2−^ + M^n+1^ (3)

SO_4_•^−^ + H_2_O → SO_4_^2−^ + OH• + H^+^ (4)

SO_4_•^−^ + OH^−^ → SO_4_^2−^ + OH• (5)

The advantages of persulfate oxidation make it an ideal candidate for coupling with bioremediation, and this strategy has been successfully applied for petroleum contaminants such as total petroleum hydrocarbons (TPHs) in diesel [41,42,43], PAHs [44,45,46,47], and BTEX [48,49,50]. To date, no studies have examined coupling persulfate oxidation and biodegradation for NA removal and OSPW toxicity reduction. Persulfate oxidation alone for NA removal has demonstrated positive results using activation methods such as ultraviolet (UV) [51,52,53,54], heat [55,56,57], and iron [38,58]. In addition, activated persulfate has been shown to be more effective than hydrogen peroxide and chlorine [51,54].

Some studies have suggested that unactivated persulfate is not reactive with NAs [51,52]. However, the short time frame of these studies (minutes–hours) would be insufficient for an unactivated persulfate reaction, which requires anywhere from days to months [38,59]. Despite the longer timeframes involved, unactivated persulfate has the advantage of being more economical, having fewer scavenging reactions, and being less harmful to microbial populations. Furthermore, longer timelines are ideal for oil sands in situ applications such as end pit lakes and groundwater systems.

The potential for unactivated persulfate oxidation coupled with bioremediation for NA removal has not yet been studied and is a primary focus of this research project. The objectives of this study were to: (1) compare persulfate oxidation alone with persulfate oxidation coupled to biodegradation for NA removal and toxicity reduction; (2) investigate the performance of unactivated (21 °C) persulfate compared to heat-activated (30 °C) persulfate; (3) determine the concentration of persulfate that improves biodegradation potential with the least detrimental effect on microbial viability.

## 2. Materials and Methods

### 2.1. Source of NAs and Bacteria

Commercial NAs were gifted from Merichem Chemicals and Refinery Services LLC (Houston, TX, USA). Merichem NAs are known to only have O_2_^−^ species and are the closest match to NAFC in OSPW [60] and, thus, were used to create a controlled system focused on the most toxic NA fraction.

*Pseudomonas fluorescens* was chosen to study biodegradation potential, as it is a well-known hydrocarbon degrader that is native to OSPW and has previously shown potential to degrade NAs [61,62]. *P. fluorescens* LP6a was previously obtained from Dr. Julia Foght and had been isolated from an enrichment culture derived from petroleum condensate-contaminated soil [63,64]. Preliminary unpublished work in our lab verified that *P. fluorescens* LP6a is capable of degrading BTEX compounds and grows in the presence of both Merichem NAs and OSPW NAFCs.

### 2.2. Experimental Setup

Unless otherwise stated, all materials were purchased from Thermo Fisher Scientific (Waltham, MA, USA). Microcosms were created using 1 L Fisherbrand^TM^ glass media bottles, filled aseptically with 500 mL of Bushnell Haas (BH) media (per L: 1 g K_2_HPO_4_; 1 g KH_2_PO_4_; 0.2 g MgSO_4_·7H_2_O; 0.02 g CaCl_2_·2H_2_O; 0.05 g FeCl_3_; 1 g NH_4_NO_3_, pH adjusted to 8.2), 100 mg/L Merichem NAs, and either 0 (biological control), 100, 250, 500, or 1000 mg/L of sodium persulfate (>98%, Sigma Aldrich). Bottles were shaken at 150 rpm. For the first stage of the experiment, bottles were loosely topped with aluminum foil to prevent bacterial contamination, while allowing for the release of carbon dioxide produced from the persulfate reaction, thus mitigating potential pH drops. Unactivated persulfate (P) was tested at room temperature (21 °C) and heat-activated persulfate (AP) at 30 °C.

The chemical reaction between Merichem NAs and persulfate was left for approximately 2 months (68 days for unactivated persulfate, 52 days for activated persulfate) to provide time for the persulfate to oxidize NAs into more bioavailable carbon sources before the *P. fluorescens* (PF, APF) was inoculated with 6.0 × 10^5^ CFU/mL. The persulfate reaction was not quenched in order to better mimic in situ conditions and examine any stress effects on the bacteria from the combination of oxidant and contaminant, creating the coupled treatment bottles (100PF–1000PF, 500APF–1000APF; Table 1). Fisherbrand^TM^ caps, modified with 20 mm blue butyl septa, were added at the same time as *P. fluorescens* to allow for the measurement of headspace carbon dioxide (CO_2_ (g)). pH was routinely monitored and adjusted to 8.2 by the addition of 1.5 mL of 1 N NaOH when required (data not shown).

Negative controls contained only 100 mg/L of Merichem NAs (0P), biological control bottles did not have persulfate added (0PF, 0APF), and chemical control bottles were not inoculated with bacteria (100P–1000P, 500AP–1000AP) in order to differentiate the chemical and biological contribution in the coupled bottles. No carbon controls (NC) were created containing 1000 mg/L of persulfate without Merichem NAs to determine the decomposition of persulfate in the absence of NAs. Treatments containing *P. fluorescens* were set up in triplicate, and chemical controls were set up in duplicate (Table 1).

### 2.3. Merichem NAs

The persulfate reaction was quenched with excess sodium thiosulfate, filtered with 0.22 µm nylon filters, and stored at 4 °C until analysis. Merichem NA content in samples was then determined using Fourier transform infrared (FTIR) spectroscopy [30,65,66]. Briefly, 8 mL of filtered samples were acidified to pH 2.0 by adding concentrated HCl, exchanged three times with approximately 5–6 mL of HPLC grade dichloromethane (DCM), passed through a sodium sulfate column to remove trace water, and then dried overnight. The dried Merichem NAs were then reconstituted in a known amount of DCM, and measurement was completed on a spectrum 100 FTIR (Perkin Elmer, Waltham, MA, USA) using a 3 mm-spaced, potassium bromide window cell (International Crystal, Garfield, NJ, USA). Data were collected with Spectrum software (V10, Perkin Elmer, Waltham, MA, USA) using 32 scans with a spectral resolution of 4 cm^–1^. Absorbance at 1743 cm^–1^ (for monomers) and 1706 cm^–1^ (for hydrogen-bonded dimers) was summed to calculate total Merichem NAs concentration. Standard solutions were prepared using known concentrations of Merichem NAs (Merichem Chemicals and Refinery Services, Houston, TX, USA) in DCM.

### 2.4. Chemical Oxygen Demand

Chemical oxygen demand (COD) was measured with HACH COD low-range (LR; limits of 3–150 mg/L) digestion solution vials (product 2125815, Fisher Scientific, Fair Lawn, NJ, USA) following method 8000, using a HACH digestor reactor and UV-Vis Hach DR/4000 spectrophotometer (Hach, Loveland, CO, USA). COD was measured immediately after sampling, without filtering or quenching samples.

### 2.5. Carbon Dioxide

Headspace CO_2_ (g) was measured with an Agilent 7890A gas chromatograph equipped with a thermal conductivity detector (GC-TCD) (Agilent HP-PLOT/Q column: 30 m × 320 µm × 0.2 µm) using an injection volume of 100 µL. The oven temperature gradient was as follows: 50 °C for 2 min, then increased at a rate of 30 °C min^–1^ to 150 °C, which was maintained for 2 min. Helium was used as carrier gas with the following flow program: 8.83 mL min^–1^ for 2 min, decreasing to 5.67 mL min^–1^ until the end of the separation. Total run time was 7.33 min. The detector was maintained at 200 °C and the injection port at 300 °C. The makeup gas (helium) was set to 5 mL min^–1^. The injector split ratio was set to 5:1 (no gas saver), with a column flow of 8.89 mL min^–1^, split vent flow of 44.4 mL min^–1^, and a septum purge flow of 58.3 mL min^–1^ under a pressure of 30 pounds per square inch (psi). Standards were created using various concentrations of CO_2_ and N_2_ gas mixes in Tedlar gas sampling bags.

### 2.6. Persulfate

The persulfate (S_2_O_8_^2–^) concentration in solution was determined based on a rapid spectrophotometric method developed by Liang et al. [67]. In this method, 500 µL of sample was added to a 40 mL EPL glass vial containing 0.2 g of NaHCO_3_, 4 g of KI, and 40 mL of Milli-Q water. The resulting solution was hand-shaken and allowed to equilibrate at room temperature for 15 min. The absorbance of 1 mL of solution at 352 nm was then measured on a UV-Vis spectrophotometer (Nano Drop 2000c Spectrophotometer, Thermo Fisher Scientific, Waltham, MA, USA).

### 2.7. Bacterial Enumeration

Viable bacterial cell counts were obtained using the drop plate method [68]. A series of 10-fold dilutions were performed to obtain the dilution that would provide 30–300 colonies per plate. One hundred microliters of each dilution was plated on Luria–Bertani (LB) agar plates (per L: 10 g peptone; 5 g yeast extract; 5 g sodium chloride; 12 g agar) in triplicate. Plates were incubated at 28 °C for 24 h before counting.

### 2.8. Microtox Assay

Acute toxicity was measured immediately after sampling (samples were not filtered or quenched) at the beginning and end of the experiment, via a Microtox assay with a Model 500 Analyzer (Modern Water, New Castle, DE, USA) and Microtox Omni 4.3 (Modern Water, New Castle, DE, USA) software. The 81.9% basic toxicity method was employed with four 2:1 dilutions and incubation times of 5 min and 15 min. There was no significant difference between incubation times; therefore, only the 5 min data were used to estimate the IC_50_ toxicity, which represents the percent volume of sample required to reduce the bioluminescence of the test specimen (*Vibrio fischeri*) by 50%. Toxicity units (TU) were then derived from IC_50_ (TU = 100/IC_50_). Phenol (100 mg/L) and deionized water were used as positive and negative controls, respectively, according to the manufacturer instructions (data not shown).

### 2.9. Statistical Analysis

Welch’s *t*-tests (two-sample unequal variance) were performed in Microsoft Excel 2014 to examine the statistical significance of the results. *p* values less than 0.05 were used to indicate a statistically significant difference.

## 3. Results and Discussion

To determine the efficacy of coupling persulfate oxidation and biodegradation for NA remediation, the degradation of organics, including both Merichem NAs and by-products, was first investigated; second, the impact of the coupled treatment system on the bacteria and subsequent toxicity reduction was examined. For each result, the coupled treatment was compared to the chemical and biological control bottles, and the effect of temperature and persulfate concentration was studied.

### 3.1. Degradation of Organics

#### 3.1.1. Merichem NA and COD Removal

**Figure 1:**
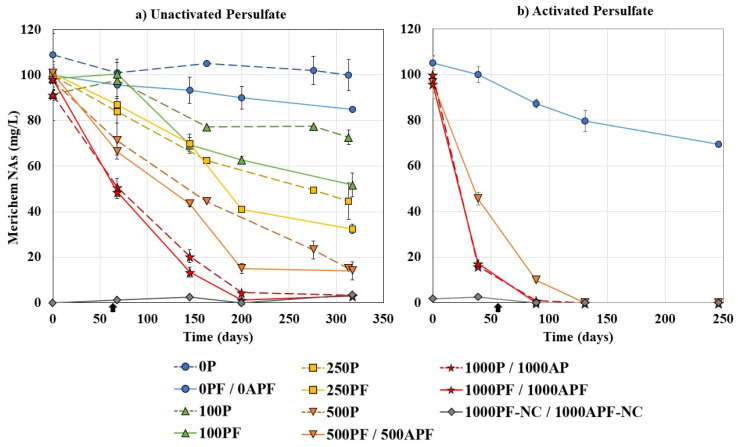
Merichem naphthenic acid (NA) concentration for (**a**) unactivated persulfate bottles at 21 °C and (**b**) activated persulfate bottles at 30 °C. Note: “P”: unactivated persulfate concentration, “PF”: unactivated persulfate coupled treatment bottles, “AP”: activated persulfate concentration, “APF”: activated persulfate coupled treatment bottles, “NC”: no carbon controls, *P. fluorescens* was added to PF bottles after 2 months (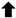), dashed lines represent chemical control bottles. All data points are an average of duplicate or triplicate bottles (Table 1). Error bars represent ±2 standard errors of the averaged value.

**Figure 2:**
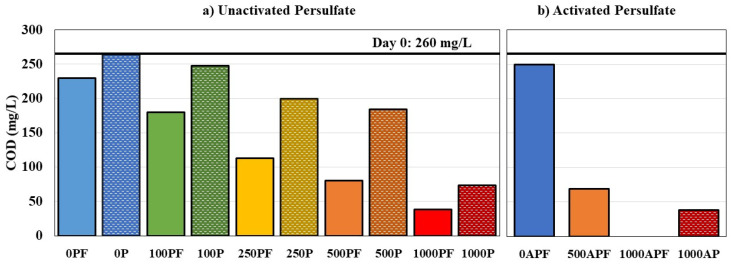
Chemical oxygen demand (COD) for (**a**) unactivated persulfate bottles at 21 °C on day 310 and (**b**) activated persulfate bottles at 30 °C on day 240. Note: “P”: unactivated persulfate concentration, “PF”: unactivated persulfate coupled treatment bottles, “AP”: activated persulfate concentration, “APF”: activated persulfate coupled treatment bottles, dashed pattern represents chemical control bottles. Data points represent a single microcosm for each condition.

NAFCs represent a major toxic fraction in OSPW; in particular, the classical O_2_^−^ species are considered main contributors to acute toxicity [6,69]. Therefore, the use of commercial NA mixtures such as Merichem NAs, which comprise solely of O_2_^−^ species, creates a controlled system to study the most toxic fraction directly, while also accounting for the complexity of a mixture of compounds [70]. Since quantifying classical NAs requires methods with high resolving power, using FTIR spectroscopy to measure the acid extractable NAFCs is commonly used in the oil sands industry [28,71]. Figure 1 displays the results of Merichem NA degradation for the unactivated (21 °C) and activated (30 °C) persulfate experiments. Chemical oxygen demand is also commonly used to determine the amount of oxidizable organics, including potential by-products without carboxyl groups that are not captured by FTIR (Figure 2).

At 21 °C, negative control bottles without persulfate or *P. fluorescens* (0P) showed no significant Merichem NA or COD removal over the course of the experiment (*p* > 0.05), indicating that degradation in experimental bottles occurred only due to persulfate oxidation and/or microbial degradation. Biological control bottles were used to demonstrate the capacity of the isolate to biodegrade Merichem NAs and, thus, contained *P. fluorescens* without persulfate (0PF). Results showed that *P. fluorescens* alone at 21 °C removed 15.9 ± 0.7% of Merichem NAs by day 313 (Figure 1a) and 11.5% of COD by day 310 (Figure 2a). Similarly, one study found that *P. fluorescens* (LD2) isolated from wetland sediments exposed to OSPW degraded only 15% of commercial Kodak NAs over 28 days; however, a mixture of *P. fluorescens* and *P. putida* degraded >95% [62]. Microbial isolates commonly do not perform as well as a microbial community where synergistic relationships exist among microorganisms [72]. In this study, an isolate was used to create a controlled system for proof of concept, and it is important to note that the treatment will likely improve when using an indigenous microbial consortium, which should be investigated in the future. However, BH media was used in this study to examine the potential of Merichem NAs to act as a carbon source without nitrogen and phosphorous as confounding variables; therefore, microbial degradation in nutrient-limited OSPW may be hindered and, thus, require biostimulation [73,74,75]. In addition, Merichem NAs generally contain linear lower-molecular-weight compounds with 7–17 carbon atoms (*n*), which are considered more biodegradable than the complex mixture of NAFCs found in OSPW including higher-molecular-weight (*n* = 7–28) compounds [5,76]. At 30 °C, the bacteria performed significantly better (*p* < 0.05), removing 33.9 ± 1.0% of Merichem NAs over a slightly shorter period of 246 days. This is expected, as *P. fluorescens* is a mesophilic microorganism with optimal growth temperatures between 25–30 °C [77].

Chemical control bottles were used to determine the capability of persulfate alone to oxidize Merichem NAs. As expected, increasing the concentration of persulfate led to increased Merichem NA removal. For unactivated persulfate (21 °C), Merichem NA removals were 30.2 ± 0.1%, 53.9 ± 10.6%, 84.5 ± 2.4%, and 95.1 ± 0.1% for 100P, 250P, 500P, and 1000P, respectively, by day 317 (Figure 1a). Persulfate concentrations of 250–1000 mg/L exhibited a linear degradation trend; however, microcosms with 100 mg/L of unactivated persulfate demonstrated a different trend, where no significant Merichem NA removal was seen in the first 68 days, followed by a large reduction at day 163, and then, no further significant removal for the remainder of the experiment (*p* > 0.05). Future work should aim to elucidate why 100 mg/L of persulfate oxidation stalled. Removal of COD was also found to increase with increasing persulfate concentration, with 4.6%, 23.1%, 29.2%, and 71.9% COD removed for 100P, 250P, 500P, and 1000P, respectively, by day 310 (Figure 2a). The incomplete COD removals suggest that the persulfate alone was mineralizing some but not all of the Merichem NAs. In particular, the lower persulfate concentrations tested (100–500 mg/L) showed very little COD removal, despite 30–85% of Merichem NAs being removed.

Overall, all chemical control bottles showed removal of organics over the course of the experiment, demonstrating that lower concentrations of unactivated persulfate than previously studied (2–10 g/L) can degrade Merichem NAs. Current literature on NA oxidation using unactivated persulfate is lacking and provides inconsistent results regarding the reaction timeframe. Sohrabi et al. [59] utilized a much higher dose of 10 g/L of unactivated persulfate and found OSPW NAFCs were removed to <1 mg/L by day 111, with the majority removed by day 60. Conversely, Drzewicz et al. [38] found that only 2000 mg/L of unactivated persulfate was needed to remove >95% of classical NAs after only 6 days. Regardless, these studies verify that unactivated persulfate can degrade OSPW NAFCs and, therefore, should be considered as a treatment option for OSPW.

At 30 °C, 1000 mg/L of activated persulfate (1000AP) led to improved Merichem NA removals, in terms of both quantity and rate, with 99.2 ± 0.6% Merichem NA removal by day 89 and 100% removal by day 131 (Figure 1b). Activating persulfate is well known to increase removal efficiency. For various model NA compounds, temperatures as high as 80 °C were required to achieve complete mineralization (100% total organic carbon (TOC) removal) in 4 h, compared to less than 10% TOC removal over 24 h at 40 °C [55]. For OSPW NAFCs, increasing the activation temperature to 80 °C led to >90% removal in 2 h compared to 6 days required at 20 °C, while activating persulfate using zero valent iron significantly improved NAFC removal at lower temperatures [38]. Likewise, 95% NA degradation occurred in only 10 min in produced water samples using 5000 mg/L of iron-activated persulfate at 23 °C [58]. Commercial Sigma NAs were found to be less reactive and only 46% were removed, indicating activated persulfate may be more reactive towards OSPW NAFC compared to commercial NA mixtures [58]. Ozonation also appears to be more effective for OSPW NAFCs compared to commercial Merichem NAs [18]. How this applies to unactivated persulfate reactivity with OSPW NAFC is uncertain. Unactivated persulfate has been shown to preferentially react with *n* = 12–14 and O_2_^−^ species in OSPW NAFCs [59], which are present in Merichem NAs, compared to activated persulfate, which is more reactive towards higher carbon numbers and ring containing NAFCs not found in commercial NA mixtures [52,54].

It is important to note that the controlled system utilized in this study (BH media and commercial Merichem NAs) may impact the persulfate oxidation kinetics compared to OSPW NAFCs. The high concentration of bicarbonate ions in OSPW have the potential to act as oxidant scavengers by forming relatively non-reactive free-radical sinks or positively contribute to degradation by propagating free-radical chain reactions [78]. Xu et al. [47] found that 0.5–10 g/L of bicarbonate had a negligible effect on model NA degradation by heat-activated persulfate. However, chloride ions can also act as oxidant scavengers and have been shown to negatively impact model NA degradation by persulfate in concentrations ranging from 0.8 to 50 g/L [47,52]. BH media contains only 0.3 g/L of chloride, while OSPW commonly contains up to 0.6 g/L [3], which may affect persulfate oxidation.

Coupled treatment bottles where *P. fluorescens* was inoculated into already reacting persulfate bottles were used to determine if the combined strategy would improve removals compared to the biological and chemical treatments alone. At 21 °C, unactivated persulfate coupled with *P. fluorescens* removed 52.8 ± 4.3%, 65.1 ± 1.1%, 85.1 ± 5.1%, and 98.9 ± 0.8% for 100PF, 250PF, 500PF, and 1000PF, respectively, by day 317 (Figure 1a). As described in Section 2.2, *P. fluorescens* was inoculated at day 68 to allow time for the persulfate to create more bioavailable carbon sources and, thus, shorten the bacteria’s lag phase. At all concentrations of persulfate tested, there was increased removal in the coupled treatments compared to the chemical control bottles. However, only 100PF exhibited significantly improved Merichem NA degradation, removing 1.8× more Merichem NAs than persulfate oxidation alone (*p* < 0.05).

An interesting trend was noted for 250PF and 500PF, where the coupled treatment bottles followed the same linear trend as the chemical control bottles until day 200, at which point the coupled treatments exhibited a sharp increase in Merichem NA removal. Therefore, while the final Merichem NA removals were not significantly different between coupled treatments and chemical control bottles (*p* > 0.05), the coupled bottles achieved the final removals more quickly.

Overall, as the concentration of persulfate was increased, the difference between chemical control bottles and coupled treatments decreased, suggesting that the persulfate was primarily responsible for the Merichem NA degradation. However, more notable differences can be seen when comparing the COD removals. At day 310, 30.8%, 56.5%, 69.2%, and 85.4% of COD was removed, for 100PF, 250PF, 500PF, and 1000PF, respectively (Figure 2a), which is 1.2–6.7× more than the chemical control bottles. This indicates that while the persulfate may have been primarily responsible for degrading the Merichem NAs, the *P. fluorescens* assisted in the removal of the oxidation by-products leading to more complete removal. Similar to Merichem NA removals, the greatest difference in COD removal between the chemical control and coupled treatments bottles (6.7×) was seen at the lowest concentration of persulfate tested (100 mg/L).

As with the chemical control bottles, the coupled system at 30 °C removed more Merichem NAs at a faster rate, with 99.7 ± 0.3% removed by day 131 for 500APF, and 99.4 ± 0.3% removed by day 89 for 1000APF. Even with >99% Merichem NA removal, 500APF exhibited 73.5% COD removal, which is only 1.1× more COD removed compared to the coupled system at 21 °C (500PF). The only treatment that resulted in 100% removal of COD was 1000APF, possibly due to the sulfate radicals assisting the *P. fluorescens* with degrading by-products.

This trend where biodegradation enhances overall removal by assisting with by-product degradation is comparable to studies that have coupled ozonation with biodegradation for OSPW remediation. Brown et al. [30] found that ozonation removed OSPW NAFCs but did not decrease dissolved organic carbon (DOC) and, instead, doubled the labile fraction of DOC allowing for more complete mineralization by microorganisms. Dong et al. [28] also noted that ozonation alone and combined with biodegradation removed similar amounts of OSPW NAFCs (33.6–41.5%); however, there was a significant difference in COD, with ozone alone removing only 13.5%, while the combined system with biodegradation removed 62.5%. Ozonation appears to remove more COD for certain commercial NA mixtures, as Vaiopoulou et al. [27] found 50% COD removal of NAs obtained from Tokyo Chemical Industry Co. (TCI); however, the removal further increased to 89% removal after biodegradation.

Coupled persulfate oxidation and biodegradation has not previously been studied for OSPW remediation but has shown positive results for other common environmental contaminants. For diesel contaminated soil, Bajagain et al. [42] found that application of a 50 mN persulfate foam followed by 1 month of bioaugmentation removed 1.2× more TPHs than the persulfate foam alone and 1.5× more than when only bioaugmented. Some studies have suggested persulfate provides a biostimulatory effect by releasing nutrients. In particular, lower persulfate concentrations have been recommended to utilize the enhanced removal without the negative effects on physicochemical and biological soil properties [44]. Medina et al. [46] found that persulfate (33 g/kg) removed a significant amount of PAHs and created more bioavailable daughter compounds, while also oxidizing and mobilizing organic matter from the soil matrix, promoting cometabolic degradation. This coupled system saw a substantial increase in aliphatic hydrocarbon removal to 66%, compared to negligible removal for persulfate or biodegradation alone, and improved PAH removal by 1.6× compared to persulfate oxidation alone.

This is the first study to couple persulfate oxidation and biodegradation for oil sands remediation and demonstrated up to 1.8× improvement in Merichem NA removal and 6.7× for COD removal compared to persulfate oxidation alone. While COD can be used as a general indicator of mineralization, it is important to examine the amount of CO_2_ produced as an ideal non-harmful end-product.

#### 3.1.2. CO_2_ Production

**Figure 3:**
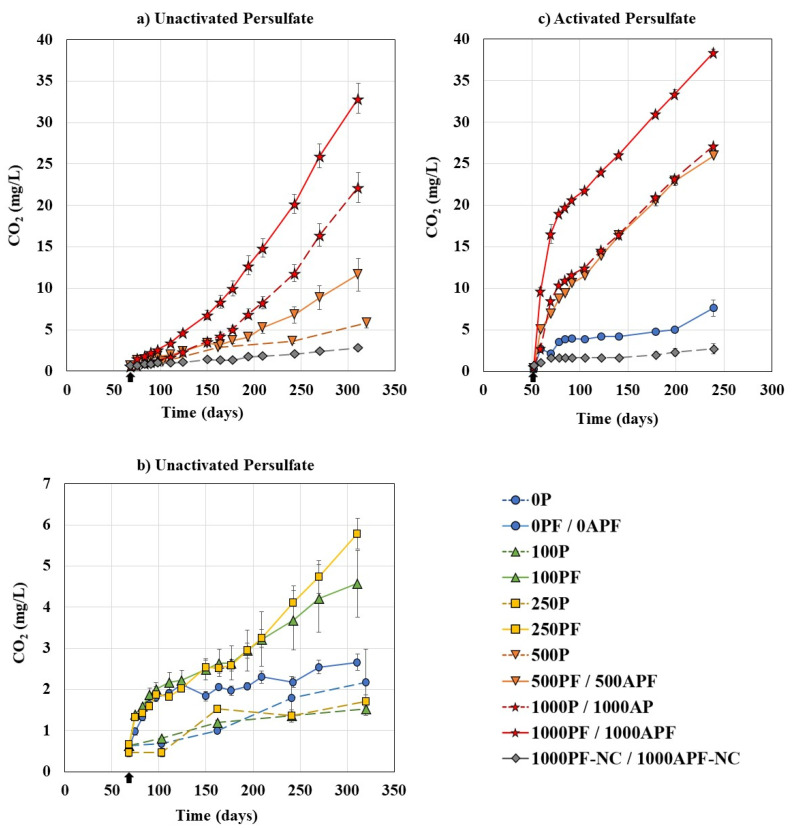
Carbon dioxide concentration in the headspace for (**a**,**b**) unactivated persulfate bottles at 21 °C and (**c**) activated persulfate bottles at 30 °C. Note: “P”: unactivated persulfate concentration, “PF”: unactivated persulfate coupled treatment bottles, “AP”: activated persulfate concentration, “APF”: activated persulfate coupled treatment bottles, “NC”: no carbon controls, *P. fluorescens* was added to PF bottles after 2 months (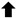), dashed lines represent chemical control bottles. All data points are an average of duplicate or triplicate bottles (Table 1). Error bars represent ±2 standard errors of the averaged value.

Carbon dioxide production in the headspace can be used to estimate the mineralization of organics and track microbial growth as a metabolic by-product. After approximately 2 months of persulfate oxidation (68 days for unactivated, 52 days for activated), *P. fluorescens* was inoculated into coupled treatment bottles, lids were added to close the system to air, and CO_2_ (g) was tracked (Figure 3).

Biological control bottles containing *P. fluorescens* alone (0PF) showed a gradual increase in CO_2_ to a final concentration of 2.7 ± 0.2 mg/L, suggesting some microbial growth and mineralization of organics; however, the increase was not statistically significant (*p* > 0.05) compared to control bottles without bacteria (0P). At 30 °C, *P. fluorescens* produced more CO_2_, demonstrating a slow increase to day 199 before jumping to 7.6 ± 0.99 mg/L by day 240. The increase in CO_2_ production at 30 °C corresponds with the greater extent of Merichem NA removal (Figure 1b).

Chemical control bottles at 21 °C produced 1.5 ± 0.07, 1.7 ± 0.2, 5.9 ± 0.6, and 22.2 ± 1.8 mg/L CO_2_ by day 320, for 100P, 250P, 500P, and 1000P, respectively (Figure 3). Both 100P and 250P did not produce significantly more CO_2_ than the negative control bottles (0P) (*p* > 0.05), indicating that very little Merichem NAs were mineralized at this concentration, which is in line with the small COD decrease noted above (Figure 2a). Conversely, 500P and 1000P produced 2.7× and 10.1× more CO_2_ than the negative control bottles, suggesting that persulfate was able to mineralize the Merichem NAs. In general, ozonation transforms rather than mineralizes NAFCs, causing ozone persistent NAFCs and by-products to accumulate [18,27]. Conversely, the results of this study suggests that persulfate does mineralize Merichem NAs. Similarly, Sohrabi et al. [59] found that persulfate led to a more complete mineralization of OSPW NAFCs, seen by 100% and 94% removal of NAFCs and DOC, compared to permanganate, which, despite removing 93% of NAFCs, saw only 33% DOC removed.

Coupled treatments with *P. fluorescens* and persulfate at 21 °C (100PF−1000PF) all exhibited significantly more (*p* < 0.05) CO_2_ production compared to their respective chemical control bottles. At day 311, there was 4.6 ± 0.8, 5.8 ± 0.4, 11.6 ± 1.9, and 32.9 ± 1.8 mg/L of CO_2_ produced, for 100PF, 250PF, 500PF, and 1000PF, respectively (Figure 3a,b). Similar to the Merichem NA degradation results (Figure 1a), the lower concentrations of persulfate exhibited a larger difference between chemical controls bottles and coupled treatments; in particular, 3.4× more CO_2_ was produced for 250PF compared to 250P. Conversely, as the concentration of persulfate increased, the difference between chemical control and coupled bottles declined, with 500PF producing 2.0× more than 500P, and 1000PF producing only 1.5× more CO_2_ than 1000P. Potentially, the persulfate reaction was more aggressive at higher concentrations and preferentially reacted with Merichem NAs and by-products, decreasing the carbon sources available for *P. fluorescens* in addition to higher oxidative stress. Regardless, the overall enhanced CO_2_ production and COD removal noted for coupled treatments suggests that there was increased Merichem NA mineralization with the addition of *P. fluorescens*.

The most CO_2_ was produced at 30 °C for 1000APF (38.4 ± 0.34 mg/L), in addition to the complete removal of COD noted earlier, this indicates complete mineralization of all Merichem NAs for this condition.

#### 3.1.3. Persulfate Persistence

Unactivated persulfate is known to be a persistent oxidant, which is a significant advantage in situ, as it allows time for the oxidant to come in contact with the contaminant. Yen et al. [79] demonstrated that unactivated persulfate could persist for 5 months in diesel contaminated soil, and Li et al. [80] found that iron-activated persulfate had a longer half-life compared to hydrogen peroxide and permanganate for benzene degradation, only decreasing 35% over 40 days.

**Figure 4:**
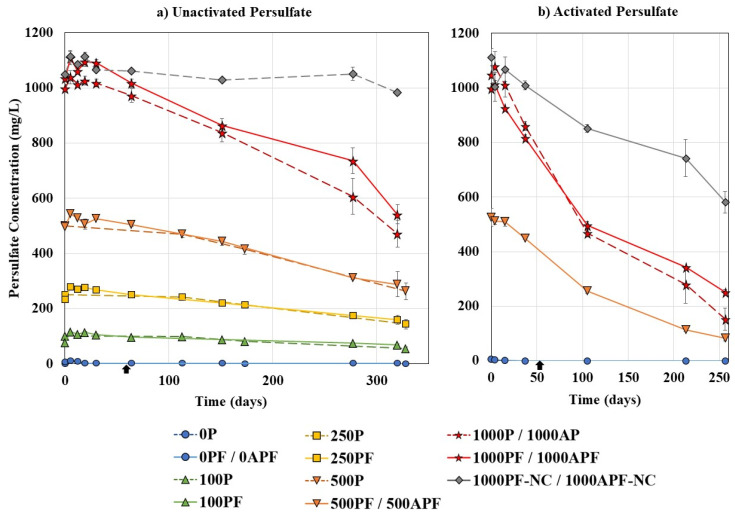
Persulfate concentration for (**a**) unactivated persulfate bottles at 21 °C and (**b**) activated persulfate bottles at 30 °C. Note: “P”: unactivated persulfate concentration, “PF”: unactivated persulfate coupled treatment bottles, “AP”: activated persulfate concentration, “APF”: activated persulfate coupled treatment bottles, “NC”: no carbon control, *P. fluorescens* was added to PF bottles after 2 months (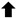), dashed lines represent chemical control bottles. All data points are an average of duplicate or triplicate bottles (Table 1). Error bars represent ±2 standard errors of the averaged value.

Figure 4 illustrates the change in persulfate concentration over the experimental timeframe. Regardless of the concentration of persulfate added and the amount of Merichem NAs removed, over 320 days at 21 °C, the persulfate proved to be persistent and had approximately half remaining in the system (45.6–61.7%). Sohrabi et al. [59] found persulfate was persistent in OSPW with less than 10% of 10 g/L of persulfate consumed over 111 days.

Chemical control bottles and coupled treatment bottles did not show a statistically significant difference in persulfate removal (*p* > 0.05). The no carbon control (1000PF–NC) was used to demonstrate the decomposition of persulfate in the absence of Merichem NAs and had 88.0 ± 0.9% remaining (982.1 ± 9.6 mg/L) by day 320. It is possible that the persulfate was reacting with the small concentration of organics from the inoculated cells and that there was some level of activation even at 21 °C, causing decomposition of the persulfate anion [78].

Based on the stoichiometric COD model proposed by Mahour [81], to remove 260 mg/L of COD (the equivalent of 100 mg/L Merichem NAs) would require a dose of 3.9 g/L of persulfate. Therefore, 100–1000 mg/L of persulfate should theoretically remove 2.6–26% COD. As discussed earlier, at 21 °C, 100–1000 mg/L of persulfate alone removed 30.2–71.9% COD, considerably more than predicted, with half the persulfate remaining in solution. Therefore, it is likely that sulfate radicals were created even at 21 °C, and radical chain reactions enhanced removal. Future work should monitor the presence of radicals to verify the presence and impact of radicals at room temperature.

Raising the temperature to 30 °C activated the persulfate and caused increasing amounts of sulfate radicals and possibly hydroxyl radicals to be created, which led to considerably faster removal of persulfate, with 14.6–25.1% remaining by day 260 (Figure 4b). A steep linear decrease was observed until day 105, at which point persulfate removal slowed, likely because there were little organics left to react with, as all Merichem NAs were removed by day 89 and 131 for 1000APF and 500APF, respectively. The creation of radicals due to heat also led to an increase in the decomposition of persulfate in the absence of Merichem NAs (1000APF–NC), with only 52.3 ± 2.0% remaining by day 260.

In OSPW studies combining ozonation with biodegradation, ozonation often does not fully remove the bio-persistent fraction leading to biodegradation activity eventually stalling [28,30]. In addition, increasing the ozone dose does not appear to lead to increased biodegradability [28]. As ozone reacts quickly, repeated applications may be required to remove the bio-persistent fraction. In this study, there was not a stall in Merichem NA degradation in coupled persulfate and biodegradation treatment bottles (Figure 1). The persistence of persulfate allowed the persulfate and *P. fluorescens* to continually act synergistically throughout the experiment duration, with the persulfate breaking down the Merichem NAs, while the bacteria degraded the by-products. Similarly, oxidant persistence has been positively correlated to diesel removal, with enhanced removal seen when using persulfate compared to permanganate and hydrogen peroxide [41].

### 3.2. Impact on Bacteria

In addition to examining the impact of the coupled treatment on removal of organics, it is important to understand the impact on the bacteria. A treatment tailored to the microorganisms is not only more cost effective, as it requires a lower quantity of chemical oxidant, but can also be quicker and lead to more comprehensive overall remediation [16,17,33,34]. This study looked at the viability of *P. fluorescens*, as the Merichem NA degrader of interest, and toxicity towards *V. fischeri* as the industry standard used to reflect toxicity towards aquatic microorganisms.

#### 3.2.1. Microbial Viability

**Figure 5:**
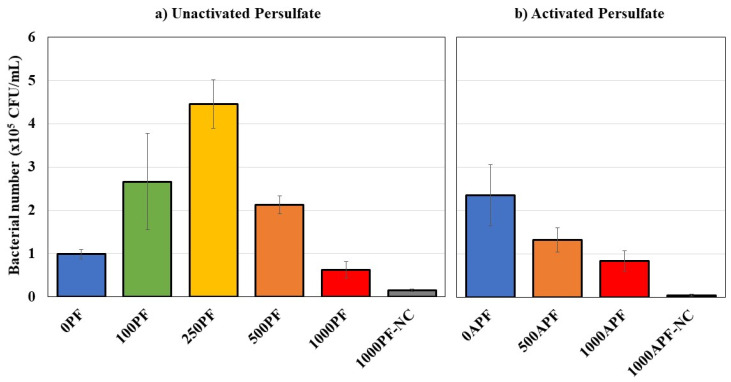
Number of colony-forming units (CFUs) for (**a**) unactivated persulfate bottles at 21 °C on day 320 and (**b**) activated persulfate bottles at 30 °C on day 260. Note: “P”: unactivated persulfate concentration, “PF”: unactivated persulfate coupled treatment bottles with *P. fluorescens*, “AP”: activated persulfate concentration, “APF”: activated persulfate coupled treatment bottles with *P. fluorescens*, “NC”: no carbon controls. All data points are an average of triplicate bottles (Table 1). Error bars represent ±2 standard errors of the averaged value.

Viable cell counts were completed to identify the number of living cells in the bottles at the end of the experiment, shown in Figure 5, which can indicate the availability of carbon sources along with potential toxic effects from the Merichem NAs and persulfate. Plate counting is known to underestimate the quantity of bacterial cells [82,83]; however, bacterial numbers have demonstrated similar trends when compared to measuring cell concentration via real-time polymerase chain reaction (qPCR) [28].

Control microcosms without Merichem NAs (1000PF-NC) did not show extensive growth, indicating that there were no other sources of carbon in the bottles. Biological control bottles containing only *P. fluorescens* (0PF) yielded 9.9 × 10^4^ ± 1.1 × 10^4^ CFU/mL at 21 °C and 2.4 × 10^5^ ± 7.0 x× 10^4^ CFU/mL at 30 °C, further verifying that *P. fluorescens* prefers 30 °C, as there were 2.4× more viable cells in addition to the improved Merichem NA degradation (Figure 1b).

Increasing concentrations of persulfate (0–250 mg/L) led to increased bacterial number until 500 mg/L, at which point they declined considerably (Figure 5). For 100PF microcosms, there were 2.7 × 10^5^ ± 1.1 × 10^5^ CFU/mL by day 320. These microcosms exhibited the greatest variability in microbial growth due to one triplicate bottle that formed more bacterial number; excluding the outlier, the average of the two remaining triplicates is 1.3 × 10^5^ CFU/mL, which is only slightly higher than the biological controls (0PF), suggesting that 100 mg/L of persulfate did not significantly enhance microbial viability.

Microcosms with 250PF produced the highest number of colony-forming units at 4.5 × 10^5^ ± 5.6 × 10^4^ CFU/mL (Figure 5), which is 4.6× the amount generated at 0PF, indicating that this concentration of persulfate provided the ideal balance between the production of more biodegradable carbon sources, while not creating significant oxidative stress. Further increasing the concentration of persulfate to 500 mg/L increased stress on the bacteria and, thus, decreased bacterial number to 2.1 × 10^5^ ± 2.0 × 10^4^ CFU/mL; however, there were still significantly more (*p* < 0.05) at 500PF compared to 0PF, likely due to the increase in bioavailable carbon sources. At 30 °C, the activated persulfate produced a higher concentration of sulfate and hydroxyl radicals that inhibited the bacteria and decreased colony-forming units to 1.3 × 10^5^ ± 2.8 × 10^4^ CFU/mL (500APF), which was 0.5× less than biological control bottles at 30 °C (0APF).

The highest concentration of persulfate tested, 1000 mg/L, exhibited the lowest bacterial number at 6.2 × 10^4^ ± 1.9 × 10^4^ CFU/mL (Figure 5). Despite the improved Merichem NA and COD removal (Figure 1a and Figure 2a), the bacteria were subject to more oxidative stress and, thus, produced 0.3× less bacterial number than at 0PF, implying that this concentration is unfavorable for *P. fluorescens.* At 30 °C, there were slightly more colony-forming units produced at 8.4 × 10^4^ ± 2.3 × 10^4^ CFU/mL for 1000APF compared to 1000PF, despite the increased stress from the radicals; however, similar to 500APF, there were still 0.4× less bacterial number than at 0APF, overall suggesting that microbial viability was not enhanced in coupled systems using activated persulfate.

Currently, only one other study simultaneously coupled chemical oxidation and biodegradation of OSPW to evaluate the impact of the oxidation on the microorganisms. Brown et al. [30] found that an ozone dose of 50 mg/L did not completely inhibit microbial growth, as ozonated OSPW showed higher *rpoB* gene copy numbers than the sterile control. However, ozonated OSPW did not show clear evidence of improved growth compared to raw OSPW. Conversely, Dong et al. [28] re-inoculated ozonated OSPW with unstressed indigenous microorganisms, which exhibited no lag phase and reached a peak of 10^6^ CFU/mL at day 60, compared to indigenous microorganisms in raw OSPW, which had a 15-day lag period and reached only 10^5^ CFU/mL by day 70.

Persulfate is generally considered less harmful to microorganisms compared to other commonly used chemical oxidants; however, the reason why has not yet been determined. Compared to potassium permanganate and Fenton’s reagent, iron-activated persulfate gave the highest PAH removal and showed the greatest increase in microbial plate counts following oxidation [36]. For benzo[a]pyrene removal, a higher concentration of iron-activated persulfate (20 mM) was able to be utilized without damaging microbial degradation compared to only 10 mM for permanganate [84]. In addition, 10–20 mM (2.7–5.4 g/L) of iron-activated persulfate increased the occurrence of PAH degrading bacteria, leading to 12–18% higher degradation efficiency compared to permanganate. Similarly, Cassidy et al. [33] found that while iron-activated persulfate alone removed the least amount of DNT, the persulfate showed a minimal impact on both number of indigenous soil microorganisms and rebound time, leading to overall DNT remediation occurring in 14 days, compared to 30 and 90 days required for Fenton’s reagent and ozone, respectively. This demonstrates that designing the treatment based on what is least harmful to the microorganisms leads to more effective overall remediation.

Tsitonaki et al. [85] studied the effect of heat-activated persulfate (40 °C) on both indigenous soil microorganisms and the isolate *Pseudomonas putida* (KT2440) using microscopic enumeration to measure cell density and acetate consumption to measure metabolic activity. Results showed that oxidation with persulfate was less damaging toward microorganisms compared to similar studies using Fenton’s reagent, hydrogen peroxide, and permanganate. Persulfate concentrations ranging from 0.1 to 10 g/L did not affect the indigenous site microbial cell density but were detrimental to *P. putida* at 10 g/L. Other studies have also shown that exponentially growing laboratory strains are more vulnerable to oxidative and heat stress compared to stationary phase cultures [86,87,88]. However, for both indigenous microorganisms and *P. putida*, acetate consumption was inhibited at 10 g/L, suggesting that membrane integrity does not always relate to metabolic activity. It is important to note that Tsitonaki et al. [85] did not adjust for pH, and at 10 g/L of persulfate, the pH dropped to 3, which likely played a role in the damaging effects at that concentration.

The reduction in pH is a disadvantage when using persulfate and is likely a primary reason for studies that noted detrimental effects in microbial density following persulfate oxidation [41,45,89,90]. In this study, pH was monitored and controlled to avoid microbial stress due to changing pH (data not shown). The unactivated persulfate bottles did not show a change in pH and did not require adjustment, while the activated bottles needed to be supplemented with sodium hydroxide 18 days after the addition of *P. fluorescens* when a closed system was created and the pH dropped to 6.5, demonstrating an advantage to using unactivated persulfate despite slower reaction rates. However, pH changes in the field are often mitigated due to incoming groundwater, and when studies on persulfate oxidation coupled to biodegradation have reported decreases in microbial density, fast rebounds to above pre-exposure levels were observed [45,46,50].

#### 3.2.2. Toxicity Reduction

The mechanism of action for NA acute toxicity is considered to be from their surfactant characteristics, which allow them to penetrate and disrupt the cell cytoplasmic membrane, altering membrane properties such as fluidity, thickness, and surface tension, leading to cell death [15,91,92,93]. However, it is important to note that OSPW has many additional organic and inorganic compounds that contribute to toxicity [4]. Furthermore, the addition of a chemical oxidation can generate more toxic by-products and act as a stressor itself, creating an increasingly complex system. To date, this is the only study that did not quench persulfate prior to toxicity measurement in order to determine the toxicity of the whole system rather than just the NAFCs.

Table 2 outlines the TU towards *V. fischeri* obtained from Microtox for the start and end of the experiment. The toxicity of 100 mg/L of Merichem NAs was found to be 11.30 TU based on the IC_50_ 5 min acute toxicity results. Comparatively, OSPW has much lower toxicity, ranging from 1.49 to 4.17 TU, based on IC_50_ [4]. As discussed previously, Merichem NAs consist solely of O_2_^−^ species, which are considered the most acutely toxic fraction and, thus, cause the discrepancy in toxicity values compared to OSPW.

Negative control microcosms containing only 100 mg/L Merichem NAs with no bacteria or persulfate (0P) saw an increase to 14.16 TU over 320 days, possibly due to evaporation and subsequent concentration of Merichem NAs. *P. fluorescens* alone in the biological control bottles decreased the toxicity by 31.0% and 38.2%, at 21 °C and 30 °C, respectively, which are relatively similarly, despite that 2.1× more Merichem NAs were removed at 30 °C (Figure 1). However, at 21 °C, the bacteria removed 3.0× more COD (Figure 2), which may include any toxic by-products from biodegradation. Further work is required to establish the by-products created during biodegradation.

The addition of persulfate caused a substantial increase in the initial toxicity of the system, to 1.33×, 1.43×, and 2.20× higher than bottles with 100 mg/L Merichem NAs alone (0P), for 100P, 250P, and 500P, respectively, at day 0 (Table 2). For 1000 mg/L of persulfate (1000P), the toxicity increased to above the detection limit, meaning that for all four dilutions tested, the bioluminescence of *V. fischeri* was reduced by more than 50%. Interestingly, microcosms containing 1000 mg/L of persulfate without Merichem NAs (1000PF-NC) showed no toxicity via Microtox (Table 2). Together, these results suggest that acute toxicity effects of persulfate are due to synergistic interactions, whereby the toxicity is enhanced beyond that of the individual compounds.

Despite the large increase in initial toxicity, chemical control bottles at 21 °C exhibited 74.5%, 93.9%, and 97.4% decreases in TU for 100P, 250P, and 500P, respectively, by the end of the experiment (Table 2). The initial toxicity of 1000P could not be determined; however, the final TU was the lowest of all the chemical control bottles at 0.02 TU (Table 2). Moreover, as discussed in Section 3.1, most of the chemical control bottles had considerable COD remaining; therefore, the large reduction in toxicity implies that the oxidation by-products produced were less toxic than the parent compound. In particular, 250P saw a substantial toxicity reduction, paired with 53.9% of Merichem NAs removed (Figure 1), only 23.1% of COD was removed (Figure 2), and an insignificant amount of CO_2_ was produced (Figure 3).

Similarly, Sohrabi et al. [59] found no detectable toxicity of NAFCs after unactivated persulfate treatment; however, the authors used extracted NAFCs, where by-products may not be extracted by DCM, and persulfate was removed from the system. When comparing UV-activated persulfate, hydrogen peroxide, and chlorine, Fang et al. [54] found that UV/persulfate showed the greatest reduction in toxicity (via Microtox). The dissolved organic matter from the three oxidants had similar elemental compositions despite vastly different toxicity reductions, suggesting toxicity originated from non-degraded organics rather than by-products produced. Another possibility is that the UV/persulfate treatment created by-products with different molecular structure or polarity, which had lower permeability though the cell membrane compared to UV/hydrogen peroxide and UV/chlorine [54].

Coupled treatments all showed improved toxicity reduction compared to the chemical control bottles, with 86.8%, 97.5%, 100%, and 100% decrease in TU for 100PF, 250PF, 500PF, and 1000PF, respectively, by the end of the experiment (Table 2). This trend has also been observed in studies with combined ozonation and biodegradation for OSPW treatment, where despite similar NAFC removals, there is a significant difference in toxicity removal with the addition of microorganisms [13,18,27]. Vaiopoulou et al. [27] noted ozonation only decreased toxicity of commercial TCI NAs by 3.3-fold, while the addition of microorganisms improved toxicity reduction to a 15-fold decrease. For OSPW, NA removals of 50–75% [18] and 90% [13] after ozonation did not reduce toxicity towards *V. fischeri*, while the inoculation of microorganisms caused a significant acceleration of toxicity removal. The lack of toxicity reduction from ozonation alone in these studies is likely because NAs are generally not mineralized after ozonation, leading to an accumulation of potentially toxic by-products, as mentioned earlier. While this study also noted that most conditions had COD remaining (Figure 2), there was notable toxicity removed, and the higher persulfate concentrations (500–1000P) exhibited a large production of CO_2_ (Figure 3), indicating mineralization was occurring. As CO_2_ is a harmless by-product, the greater extent of mineralization with persulfate oxidation compared to ozonation is a significant advantage.

At 30 °C, all persulfate concentrations tested for both chemical controls and coupled treatments (500APF, 1000AP, 1000APF) showed a completed removal of toxicity by the end of the experiment (Table 2). This is expected since these conditions also showed complete removal of Merichem NAs (Figure 1b). Similar to above, this is interesting, as there was 14.6–25.1% of persulfate anions remaining in the system (Figure 4). In addition, since the persulfate was activated, there were also sulfate radicals present, which would have been expected to impact the toxicity. It is possible that sulfate radicals require longer timelines to penetrate the cell membrane due to their relatively large size compared to hydroxyl radicals [85]. Another possibility is that the toxic effects of persulfate anions and sulfate radicals come primarily from physicochemical changes to the environment, such as pH decreases discussed above or increased salinity from sulfate ions produced during the reaction (Equation (1)).

It is interesting to note that despite the complete removal of Microtox acute toxicity noted in activated persulfate coupled treatments, microbial viability of *P. fluorescens* was negatively impacted (Figure 5b). This further suggests that toxic effects from persulfate may be due to long-term physicochemical changes to the environment, or that there was a lack of available carbon sources if the persulfate reacted with Merichem NAs and by-products faster than the bacteria could utilize them. Regardless, the bacteria preferred the lower persulfate concentrations, which should be considered when designing a treatment strategy.

## 4. Conclusions

This study has demonstrated the potential of different doses of unactivated and activated persulfate coupled with biodegradation as an efficient treatment strategy to degrade Merichem NAs and reduce toxicity. The addition of 100 mg/L of unactivated persulfate and *P. fluorescens* improved Merichem NA degradation by 1.8× compared to persulfate oxidation alone. For unactivated persulfate concentrations of 250–1000 mg/L, the coupled treatment system inoculated with *P. fluorescens* exhibited similar Merichem NA removals compared to the chemical control bottles with persulfate alone. However, there was a large difference in COD removals and CO_2_ production when *P. fluorescens* was added to the coupled system, suggesting that the persulfate was primarily responsible for Merichem NA removal, while the *P. fluorescens* degraded the oxidation by-products. The impact of the bacteria became less pronounced at higher persulfate concentrations (1000 mg/L), likely because there were less carbon sources for the bacteria and increased stressors. Microbial density was increased with the addition of 250 mg/L of unactivated persulfate, which provided enough increased bioavailable carbon sources to outweigh the oxidative stress effect of the persulfate. At 30 °C, the production of radicals created a more aggressive oxidation reaction, improving the amount and rate of Merichem NA removal; however, microbial viability was reduced. The acute toxicity was found to increase considerably due to the addition of persulfate; however, persulfate alone did not show toxicity towards *V. fischeri,* suggesting increased toxicity may be due to synergistic interactions.

As Alberta’s oil sands industry continues to expand, there is an increasing need for more effective water management and remediation methods. Coupling persulfate oxidation and biodegradation decreases the chemicals required compared to chemical oxidation alone by utilizing microorganisms to degrade the remaining compounds, thereby significantly reducing treatment costs and the toxicity of tailings wastewater.

## Figures and Tables

**Table 1:** Summary of persulfate oxidation and biodegradation experimental bottles. Note: “NAs”: naphthenic acids, “P”: persulfate concentration, “PF”: coupled treatment bottles, “AP”: activated persulfate, “APF”: activated persulfate coupled treatment bottles, “NC”: no carbon controls, *P. fluorescens* was added after 2 months (*).

Identification Label	Persulfate (mg/L)	*Pseudomonas fluorescens* *	Merichem NAs (mg/L)	Temperature (°C)	Replicates
*Unactivated Persulfate*					
0P (0 mg/L P)	0	No	100	21	2
0PF (0 mg/L P + *P. fluorescens*)	0	Yes	100	21	3
100P (100 mg/L P)	100	No	100	21	2
100PF (100 mg/L P + *P. fluorescens*)	100	Yes	100	21	3
250P (250 mg/L P)	250	No	100	21	2
250PF (250 mg/L P + *P. fluorescens*)	250	Yes	100	21	3
500P (500 mg/L P)	500	No	100	21	2
500PF (500 mg/L P + *P. fluorescens*)	500	Yes	100	21	3
1000P (1000 mg/L P)	1000	No	100	21	2
1000PF (1000 mg/L P + *P. fluorescens*)	1000	Yes	100	21	3
1000PF-NC (1000 mg/L P + *P. fluorescens* − No Carbon)	1000	Yes	0	21	3
*Activated Persulfate*					
0APF (0 mg/L AP + *P. fluorescens*)	0	Yes	100	30	3
500APF (500 mg/L AP + *P. fluorescens*)	500	Yes	100	30	3
1000AP (1000 mg/L AP)	1000	No	100	30	3
1000APF (1000 mg/L AP + *P. fluorescens*)	1000	Yes	100	30	3
1000APF-NC (1000 mg/L AP + *P. fluorescens* − No Carbon)	1000	Yes	0	30	3

**Table 2:** Acute toxicity, measured as toxicity units, at the start (day 0) and end of the experiment for unactivated (21 °C; day 320) and activated (30 °C; day 260) persulfate bottles. Note: “P”: unactivated persulfate, “PF”: unactivated persulfate coupled treatment bottles with *P. fluorescens*, “AP”: activated persulfate, “APF”: activated persulfate coupled treatment bottles with *P. fluorescens*, “NC”: no carbon controls, “N/A”: not applicable. Data points represent a single microcosm for each condition.

Persulfate Concentration	Day 0 Experiment Start	Day 320		Day 260	
		P	PF	AP	APF
0 mg/L	11.30	14.16	7.81	N/A	6.98
100 mg/L	15.01	3.83	1.99	N/A	N/A
250 mg/L	16.13	0.97	0.40	N/A	N/A
500 mg/L	24.81	0.66	0	N/A	0
1000 mg/L	*Too Toxic*	0.02	0	0	0
1000 mg/L–NC	0	0	0	N/A	0

## Data Availability

The data presented in this study are available within the article.
